# A rapid RT-LAMP assay for the detection of all four lineages of Peste des Petits Ruminants Virus

**DOI:** 10.1016/j.jviromet.2019.113730

**Published:** 2019-12

**Authors:** Paulina Rajko-Nenow, John Flannery, Hannah Arnold, Emma L.A. Howson, Karin Darpel, Anna Stedman, Amanda Corla, Carrie Batten

**Affiliations:** The Pirbright Institute, Ash Road, Pirbright, Woking, Surrey, GU24 0NF, UK

**Keywords:** PPRV, RT-LAMP, Diagnostics, Outbreak, Surveillance

## Abstract

Peste des petits ruminants (PPR) is a viral disease of small ruminants that is caused by the PPR virus (PPRV) and is a significant burden on subsistence farmers across the developing world. Loop-mediated isothermal amplification (LAMP) provides cost-effective, rapid, specific and sensitive detection of nucleic acid and has been demonstrated to have field application for a range of viruses. We describe the development of a novel PPRV RT-LAMP assay utilising carefully-selected primers (targeting the N-gene) allowing for the detection of all known PPRV lineages in < 20 min. The assay was evaluated in comparison with a “gold standard” real-time RT-PCR assay using more than 200 samples, comprising samples from recent PPRV outbreaks, experimentally-infected goats, well-characterised cell culture isolates and samples collected from uninfected animals. The RT-LAMP assay demonstrated 100% diagnostic specificity and greater than 97% diagnostic sensitivity in comparison with the real-time RT-PCR assay. The limit of detection was between 0.3 and 0.8 log_10_ TCID_50_ ml^−1^ equating to a C_T_ value of 31.52 to 33.48. In experimentally-infected animals, the RT-LAMP could detect PPRV as early as 4 days post infection (dpi) - before clinical signs were observed at 7 dpi. The RT-LAMP assay can support the global PPR eradication campaign.

## Highlights

• Designed N-gene RT-LAMP using all full-genomes available on Genbank.
• The RT-LAMP showed comparable diagnostic sensitivity to the real-time RT-PCR assay.
• The limit of detection was estimated between 0.3 to 0.8 TCID_50_ ml^−1^.
• The RT-LAMP could detect PPRV as early as 4 dpi – before clinical signs were observed.
• The RT-LAMP assay can support the global PPR eradication campaign.

## Graphical abstract

**Figure:**
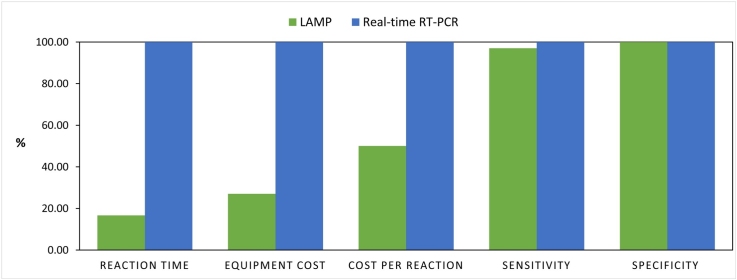

## 1 Introduction

Peste des petits ruminants (PPR) is a highly contagious disease of small ruminants that is a major obstacle to sustainable agriculture across the developing world and causes annual economic losses of 1.4–2.1 billion USD (Banyard et al., 2010; Kwiatek et al., 2011). The global burden of PPR has steadily increased since the virus was first described in Côte d’Ivoire (Gargadennec and Lalanne, 1942) and PPR now spans a range beyond western Africa to affect over 70 countries throughout Africa, the middle East and Asia (Baron et al., 2017). In 2016, PPR was first detected in Georgia at the crossroads of Western Asia and Eastern Europe (Rajko-Nenow et al., 2017). Two years later, the first incursion of PPRV into Europe was recorded in Bulgaria (OIE, 2018). A joint campaign developed by the World Organisation for Animal Health (OIE) and the Food and Agriculture Organisation (FAO) aims to control and eradicate the disease through a global strategy by 2030 (FAO, 2015).

PPR is caused by as Peste des petits ruminants virus (PPRV) belonging to the *Small ruminant morbilliviru*s species within the *Morbillivirus* genus of the family *Paramyxoviridae* (Albina et al., 2013) and is closely related to measles virus (of humans) and the now-eradicated rinderpest virus of cattle. PPRV is an enveloped virus containing a linear single-stranded negative-sense RNA genome that encodes six structural proteins: the nucleocapsid protein (N), phosphoprotein (P), matrix protein (M), fusion protein (F), haemagglutinin protein (H), large polymerase protein (L), and two non-structural proteins, C and V, generated from an alternative reading frame of the P gene (Barrett et al., 2006; Kumar et al., 2014). Although all PPRV strains belong to a single serotype, they can be classified into four distinct lineages (I to IV) based on the phylogenetic analysis of the partial N or F gene sequence (Banyard et al., 2010). PPRV lineage corresponds to the geographical distribution of the virus, with lineage I and II occurring in West Africa, lineage III in East Africa, the Middle East and southern India, and lineage IV in Asia (Dhar et al., 2002; Shaila et al., 1996).

Similar to measles virus, PPRV is highly contagious and close contact between infected and healthy animals is the main route of PPRV transmission during an outbreak (Wernike et al., 2014). Following a short incubation period of 3–10 days, the disease is characterised by fever, oculo-nasal discharge, erosive lesions in the oral cavity, respiratory problems and diarrhoea and is associated with high morbidity and mortality (Banyard et al., 2010).

Rapid and specific diagnostic assays are crucial for the control (through deployment of vaccine and movement restrictions and/or stamping out initiatives) and ultimately the eradication of this disease. A number of real-time RT-PCR assays have been developed to specifically detect PPRV (Batten et al., 2011; Polci et al., 2015; Kwiatek et al., 2010) and are used worldwide. However, real-time RT-PCR is still an expensive technique and is largely restricted to laboratory settings. This therefore presents an impediment in developing countries where equipment, trained personnel, reagent and transport costs may be beyond the resources of the veterinary services. In addition, poor infrastructure can impede sample transport to the laboratory, further delaying the implementation of appropriate control measures. There is a clear advantage in developing assays that are low-cost and can be performed in the field (i.e. pen-side). Such assays would have the potential to reduce some of the difficulties faced by developing countries in which PPR is endemic.

Reverse transcription loop-mediated isothermal amplification (RT-LAMP) is a highly sensitive nucleic acid amplification technique, which combines reverse transcriptase, a strand-displacing polymerase, and multiple primers for rapid detection of RNA. The requirement for isothermal conditions has enabled RT-LAMP to be adapted for low-cost diagnostics and use *in situ*. For instance, incubation and detection can be performed either in real-time using portable fluorimeters (Howson et al., 2017; Ashraf et al., 2017) or using end-point detection (Waters et al., 2014). Furthermore, LAMP chemistry is more resistant to inhibitors than PCR, enabling simplification of extraction procedures (Blomstrom et al., 2008; Waters et al., 2014; Howson et al., 2017). As such, RT-LAMP assays for a number of RNA viruses including bluetongue virus (Maan et al., 2016; Mohandas et al., 2015) and African horse sickness virus (Fowler et al., 2017) have been developed. An RT-LAMP assay for PPRV detection was first developed in 2010 (Li et al., 2010), but it was validated on a small sample set and the assay requires one hour incubation under isothermal conditions. Similar limitations can be found in the recently published assay by Mahapatra et al., 2019. Another PPRV RT-LAMP assay (Ashraf et al., 2017) was performed on a larger sample set which included samples from two experimentally-infected animals, however, PPRV sequences of lineage II and III were excluded from the primer design. Since the publication of these PPRV RT-LAMP assays, additional full genome sequences, representative of all four lineages, have become available in the public domain. This presents an opportunity to develop an assay using the most up-to-date sequence information available on GenBank.

Herein we describe the development of a novel PPRV RT-LAMP assay utilising carefully-selected primers allowing for the successful detection of all PPRV lineages in less than 20 min. The assay was evaluated in comparison with a “gold standard” real-time RT-PCR assay using more than 200 samples from: recent PPRV outbreaks, experimentally-infected goats, well-characterised cell culture isolates representing four PPRV lineages and samples collected from uninfected animals. In addition, the use of the PPRV RT-LAMP assay as a confirmatory field diagnostic test was investigated on ocular swabs stored at 37 °C for a number of days.

## 2 Material and methods

### 2.1 Primer design and evaluation

All full-length genome sequences of PPRV (n = 77, available in the public domain on October 2^nd^ 2018), representative of all four PPRV linages, were retrieved from GenBank and aligned using the MUSCLE algorithm in MEGA6 programme (Tamura et al., 2013). LAMP primer design was investigated against consensus sequences of gene targets F, H, L, M, P and N using LAMP Designer (OptiGene Ltd., UK). Seven initial primer sets were designed (Table S1- supplementary material), targeting regions that displayed the least sequence variation: three primer sets targeting the L gene, two primer sets targeting the F gene and two primer sets targeting the N gene.

Primer testing was performed on the L, N and F gene primer sets (sets A–G) using nucleic acid extracted from well-characterised PPRV isolates (mycoplasma free, complete genome sequence available) shown in Table 1 that were obtained through the European Virus Archive goes global (EVAg) consortium. The performance of all primer sets (A–G) was compared and the primer set that did not yield false positives and had the shortest time to positivity (t_p_) was selected for further validation.

**Table 1:** PPRV isolates used during the validation of the RT-LAMP assay. Isolates were supplied through the EVAg consortium (https://www.european-virus-archive.com).

PPRV isolate name	Lineage	Mean Titre (log_10_ TCID_50_/ml)
*PPRV Ivory Coast/1997	I	5.28
PPRV/Guinea/1988	I	4.53
PPRV/Nigeria/1975/2	II	4.89
*PPRV/Ghana/1978/1	II	5.30
PPRV/Dorcas U.A.E./1986	III	4.42
*PPRV/Iran/2011	IV	5.47
*PPRV/Georgia/Tbilisi/2016	IV	5.50

(*) Isolates used in the PPRV animal experiment.

### 2.2 RT-LAMP assay conditions

The RT-LAMP assay was performed using the Isothermal Master Mix (OptiGene Ltd UK, # ISO-004) in a 25 μl total reaction mixture volume containing: 5 μl of template, 15 μl of isothermal master mix ISO-004, 2.5 μl of 10 x primer mix, 7 U AMV reverse transcriptase (New England Biolabs, Hitchin, UK) and 1.8 μl of nuclease-free water. A working stock of 10 x primer mix contained the following primer concentrations: 2 μM of F3 primer, 2 μM of B3 primer, 20 μM of FIP primer, 20 μM of BIP primer, 10 μM of LoopF primer and 10 μM of LoopB diluted using TE buffer (10 mM Tris pH8.0).

The RT-LAMP was performed at 65 °C for 20 min using the Gene II portable fluorimeter (OpiGene Ltd, UK). Anneal analysis was performed by heating the reaction to 98 °C for 1 min, then cooling to 80 °C decreasing at 0.05 °C/s to confirm that the amplicons were PPRV-specific. The reaction time was extended to 60 min during the initial primer validation to select the best primer set.

### 2.3 Viral RNA extraction

Automated extraction of PPRV RNA was performed using 100 μl of sample (EDTA blood, cell culture isolates, tissue extracts, ocular or nasal swabs) on the Kingfisher Flex automated extraction platform (ThermoFisher Scientific, Paisley, UK) and the MagVet Universal nucleic acid extraction kit (ThermoFisher). RNA was eluted into 80 μl of elution buffer and was stored at 4 °C prior to analysis using the real-time RT-PCR assay or the RT-LAMP assay.

Manual extraction of PPRV RNA was performed on 100 μl of EDTA blood, ocular and nasal swabs using the magnetic separation rack MagnaRack (ThermoFisher) and the MagMAX CORE Nucleic Acid Purification Kit (ThermoFisher). PPRV RNA extracted from swabs was eluted into 80 μl, whereas RNA extracted from EDTA bloods was eluted into 120 μl of elution buffer. RNA was stored at 4 °C prior to analysis using the RT-LAMP assay.

### 2.4 Real-time RT-PCR

The PPRV real-time RT-PCR assay (Batten et al., 2011) was performed using the Express One-Step Superscript qRT-PCR kit (LifeTechnologies, Paisley, UK). Briefly, 3 μl of RNA (extracted using the KingFisher Flex) was added to 17 μl of one-step reaction mix containing final concentrations of 1 × reaction mix, 400 nM forward, 400 nM reverse primers, 100 nM probe, 50 nM Rox, and 2 μl of enzyme mix in each well of 96-well plate. Amplification and cycling conditions were as follows: reverse transcription at 50 °C for 15 min, RT inactivation/Taq activation at 95 °C for 20 s, and then 45 cycles of PCR, with each cycle consisting of 95 °C for 3 s, 60 °C for 30 s. Real-time RT-PCR was performed on an Applied Biosystems 7500 Fast real-time PCR instrument (LifeTechnologies).

### 2.5 Validation of the RT-LAMP assay

#### 2.5.1 In-house specificity

The specificity of the PPRV RT-LAMP assay was determined using extracted RNA derived from a range of viruses that either show close genetic relationship with PPRV e.g. measles virus (MeV), phocine distemper virus (PDV) or cause a disease that can be difficult to clinically differentiate from PPR e.g. foot-and-mouth disease virus (FMDV), bluetongue virus (BTV), sheeppox virus (SPPV) and goatpox virus (GTPV). Additionally, PPRV isolates representing four lineages (Table 1), with a titre between 4.42 to 5.50 log_10_ TCID_50_  ml^−1^ were extracted using the automated platform and tested by the RT-LAMP assay.

#### 2.5.2 Sensitivity and limit of detection

A standard curve was generated using log dilutions (10^−1^ to 10^−5^) of previously-extracted nucleic acid from four PPRV strains (Table 3). Each dilution was analysed in triplicate using the RT-LAMP assay and the real-time RT-PCR assay. For each dilution, the percentage coefficient of variation (%CV), defined as the ratio of the standard deviation (σ) to the mean, was calculated to determine the extent of variability in relation to the mean of the population. To determine the limit of detection (LOD), a 1 in 2 dilution series were created and each dilution was run in triplicate. The LOD was considered as the greatest dilution for which all replicates tested positive.

#### 2.5.3 Repeatability

The repeatability of the RT-LAMP assay was evaluated using RNA extracted from the log dilutions of the Georgia/Tbilisi/2016 strain which were tested in triplicate by three analysts (Table 5).

#### 2.5.4 Recent PPRV international outbreaks

A number of clinical samples have been submitted to the OIE Reference Laboratory for PPR at the Pirbright Institute for confirmatory testing. A total of 30 samples from recent PPRV outbreaks in three countries was extracted using the Kingfisher Flex automated platform and then analysed using the real-time RT-PCR and the RT-LAMP assays.

### 2.6 PPRV animal experiment

Goats (n = 12) aged 9–15 months were intra-nasally infected with one of four isolates of wild type PPRV (under project licence PL70/8833). Group 1 (n = 3) – Ivory Coast/1989 L N VDS1, Group 2 (n = 3) – Ghana/1978/1 LK1 Vero1 VDS1, Group 3 (n = 3) – Iran/2011 CV1-SLAM1 VDS3 and Group 4 (n = 3) – Tbilisi/Georgia/2016 IV VDS2. EDTA blood (n = 12), ocular (n = 12) and nasal (n = 12) swabs were collected from each animal before infection. EDTA blood and nasal swabs were also collected at 2 days post infection (dpi) whereas EDTA blood, ocular and nasal swabs were collected from infected goats on 4, 6, 8, and 9 dpi. Out of the 12 goats, 1 was euthanized at 7 dpi, 8 at 8 dpi and the remaining 3 at 9 dpi for reaching the humane endpoint.

EDTA blood samples were collected from a superficial vein into 2-ml purple vacutainer tubes. Ocular swabs were taken using viscose tip swabs provided with the PESTE-TEST: Rapid field Test for Peste des Petits Ruminants virus (PPRV) Infection (The Pirbright Institute, UK). The swabs were placed in ∼500 μl of PESTE-TEST buffer within 4 h after sampling. Nasal swabs were taken using cotton swabs and placed in 500 μl phosphate buffered saline (PBS) (ThermoFisher) within 4 h. All samples were stored at 4 °C and were tested within 24 h.

RNA from all samples (n = 177) was extracted using the Kingfisher Flex automated platform and then analysed using the PPRV real-time RT-PCR assay. To assess the sensitivity of the RT-LAMP assay in the field, RNA from the same samples was also manually extracted using the MagMax Core extraction kit with the magnetic separation system and then tested using the PPRV RT-LAMP assay.

Clinical observations were performed daily and the goats were assigned a score based on nasal signs (congestion = 1, discharge = 2), ocular signs (congestion = 1, discharge = 2), oral signs (redness of gums/papillae = 1, 1 or 2 vesicles in gums = 2, necrotic vesicle(s) = 3), presence of diarrhea (any = 1, watery/hemorrhagic = 2), respiration (cough = 1, noise when breathing = 2), behavior (apathetic = 1, recumbent = 2) and appetite (not eating as normal = 1, not eating = 2).

### 2.7 Detection of PPRV in ocular swabs

Ocular swabs are considered the most useful sample type to be collected in the field and are recommended for testing using the PESTE-TEST: Rapid field Test for Peste des Petits Ruminants virus (PPRV) Infection (The Pirbright Institute, UK) (Baron et al., 2014). To investigate the effect of storage conditions on the ocular swabs resuspended in the PESTE-TEST buffer, 30 μl was taken from each of the 24 goat ocular swabs between 6 and 8 dpi, pooled together and incubated at 37 °C. From this material, an aliquot of 100 μl underwent manual RNA extraction at 0, 3, 5 and 11 days post incubation. RNA extracts were stored at +4 °C before being analysed on day 11 using the RT-LAMP and the real-time RT-PCR assay. In addition, the F-gene conventional RT-PCR (Forsyth et al., 2003) using primers F1B-5′AGTACAAAAGATTGCTGATCACAGT3′, F2D-5′GGGTCTCGAAGGCTAGGCCCGAATA3′ was performed on this RNA and the PCR products were visualised using agarose gel electrophoresis to determine the suitability of these samples for Sanger Sequencing.

## 3 Results

### 3.1 Primer selection and evaluation

BLAST analysis of all primer-binding regions on representative sequences showed no significant matches to any other Morbilliviruses including rinderpest virus, measles virus, canine distemper virus and dolphin morbillivirus. Initial testing of primer sets showed that primer set A failed to detect PPRV during 60 min reaction time. Sets B and C were able to detect all PPRV samples tested (n = 7), however non-specific amplification was evident in negative samples. Primer sets E and G also displayed non-specific amplification in negative samples, so were also excluded from further analysis. Primer sets D and F were capable of detecting the four PPRV lineages, but primer set D consistently displayed a longer time to positivity. Primer set F, targeting the PPRV N-gene was taken forward for further evaluation (Table 2) as it did not generate non-specific amplicons and enabled the RT-LAMP reaction time to be reduced from 60 min to 20 min.

**Table 2:** The LAMP primers targeting the N-gene (set F) designed and validated in this study.

Oligo sequence (5' to 3')	Position	Oligo name	Final concentration in 25 μl LAMP reaction
TGTTAGCCTCCATACTAGCA	497	N_F3_f	0.2 μM
TGTCAATGTCGCAGATCATT	775	N_B3_f	0.2 μM
TGTCAAGGCGAAATTCCCCAAAGAACTGAGAAGGTGGGTTA	637; 573	N_FIP_f	2 μM
CGGCGGTTCATGGTATCTCTCCAATCCTTGGCTTGTTGC	688; 751	N_BIP_f	2 μM
TCACTCTCCTTTGTTGTGTGT	616	N_LF_f	1 μM
ATACTTGACATCAAGAGGACCC	709	N_LB_f	1 μM

**Table 3:** Intra-assay repeatability of the PPRV RT-LAMP.

Sample ID	Dilution	Mean C_T_ value	Mean T_a_ [°C]	Mean t_p_ [mm:ss]	Standard deviation σ	%CV
Ivory Coast/1997 lineage I	10^−1^	21.84	87.00	07:10	8.66	2.01
	10^−2^	25.01	87.03	08:00	0.00	0.00
	10^−3^	28.44	87.07	10:30	15.00	2.38
	10^−4^	31.63	86.90	14:05	243.87	28.86**
Nigeria/1975 lineage II	10^−1^	21.36	87.00	07:15	0.00	0.00
	10^−2^	25.51	87.03	08:20	8.66	1.73
	10^−3^	29.33	87.07	11:25	45.83	6.69
	10^−4^	32.12	86.95	13:30	150.00	18.52**
Dorcas UAE/1986 lineage III	10^−1^	20.97	86.33	08:25	8.66	1.71
	10^−2^	25.06	86.30	09:30	0.00	0.00
	10^−3^	28.96	86.27	11:40	17.32	2.47
	10^−4^*	31.65	86.30	13:15	–	-**
Iran/2011 lineage IV	10^−1^	18.91	86.90	06:40	0.00	0.00
	10^−2^	22.12	86.90	07:32	8.66	1.91
	10^−3^	26.02	86.90	08:52	0.00	0.00
	10^−4^	29.40	86.90	11:13	0.00	0.00
	10^−5^	32.67	87.00	12:40	135.28	17.80**

* Only one out of three replicates was positive.

** at or approaching the limit of the detection of the PPR RT-LAMP assay.

### 3.2 Validation of RT-LAMP assay

#### 3.2.1 In-house specificity

The RT-LAMP assay did not display cross-reactions with other viruses such as BTV, FMDV, SPPV, GTPV, MeV and PDV indicating a high degree of specificity for PPRV as shown in table S2 (Supplementary material). Extracted RNA from PPRV isolates was detected with a similar time to positivity (t_p_) and ranged from 5:30 to 8:30, well within the assay cut off time of 20 min.

#### 3.2.2 Sensitivity

**Fig. 1:**
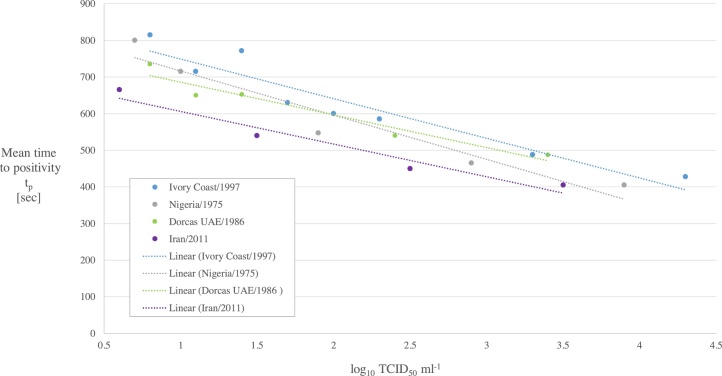
Linearity of the standard curve for PPRV RT-LAMP assay.

**Fig. 2:**
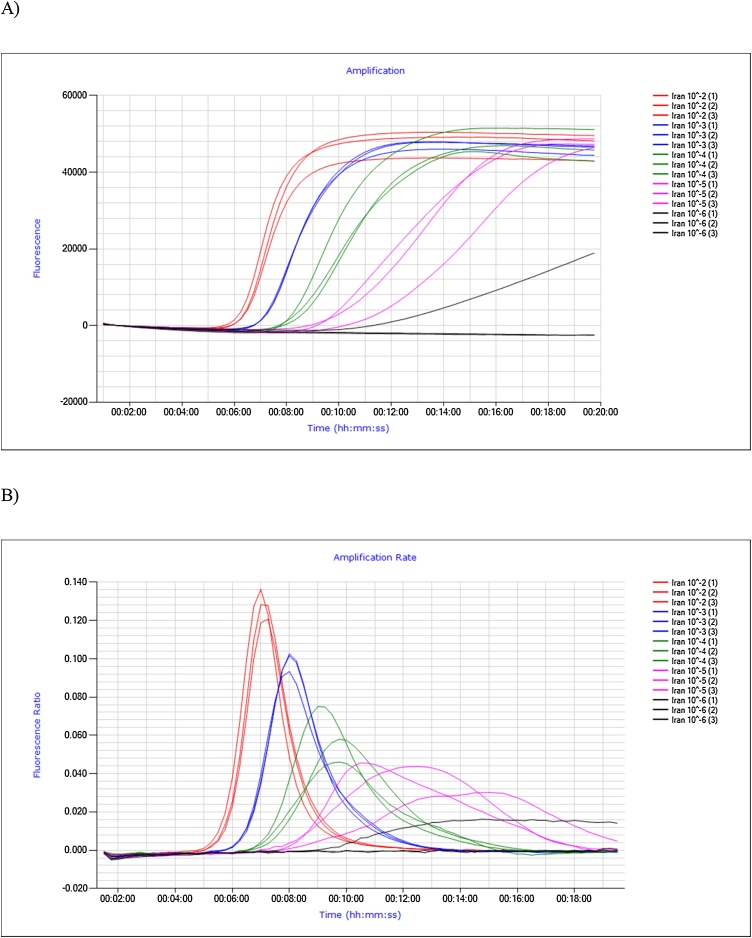
Isothermal amplification (A), amplification rate (B) for serial dilutions of PPRV Iran/2011 lineage IV.

The t_p_ obtained for each dilution was plotted against the log_10_ TCID_50_ ml^−1^ (Fig. 1) and a linear regression analysis was performed. The relationship between the mean t_p_ value and virus concentration was linear with a R-squared value (R^2^) of 0.90, 0.93, 0.94 and 0.95 for Ivory Coast/1997 lineage I, Nigeria/1975 lineage II, Dorcas UAE/1986 lineage III, and Iran/2011 IV lineage, respectively. An acceptable %CV for diagnostic assays is considered to be 10% (Reed et al., 2002) and this shows that the PPRV RT-LAMP assay meets this criterion for dilutions 10^−1^ to 10^-3^ of lineage I, II and III and for dilutions 10^−1^ to 10^-4^ of lineage IV (Table 3). A %CV greater than 10% was obtained for the greatest dilution of each strain which is considered to be close to the detection limit of the RT-LAMP assay. RT-LAMP amplification curves for log dilutions of PPRV Iran/2011 lineage IV (analysed in triplicate) are shown in Fig. 2. PPRV dilutions 10^-2^ to 10^-4^ showed fluorescence curves which were characterized by sharp peaks on the amplification rate plot (Fig. 2b), whereas the fluorescence curves for 10^-5^ replicates are shallower and irregular in shape. Dilution 10^-6^ is beyond the detection limit of RT-LAMP assay as none of the replicates were amplified.

#### 3.2.3 Limit of detection (LOD)

The LOD of the RT-LAMP assay was calculated separately for each strain representing the four PPRV lineages (Table 4) and compared with the C_T_ value of the real-time RT-PCR assay. The LOD of the RT-LAMP assay was estimated between 0.3 and 0.8 log_10_ TCID_50_ ml^−1^ which equates to a C_T_ value ranging between 31.52 to 33.48 (Table 4). When approaching the LOD of the RT-LAMP assay, the fluorescence curve became shallower and did not reach the amplification plateau phase. For each PPRV lineage in the last detectable dilution, the fluorescence curves were uneven and the t_p_ differed by a few minutes amongst replicates (Table 4). In all, the four lineages of PPRV were detected with comparable sensitivity. The mean anneal temperature (T_a_) 86.86 ± 0.30 (ranged from 85.60 °C to 87.30 °C) was calculated for all tested dilutions (10^−1^ to 10^-5^) of four lineages (Table 4).

**Table 4:** Limit of the detection of PPRV RT-LAMP assay.

PPRV strain	Log_10_ TCID_50_ ml^−1^	C_T_ value	Time to positivity t_p_ [mm:ss]	Mean T_a_ [°C] ±SD (range)
Ivory Coast/1997 lineage I	0.8	33.12	16:00 14:45 10:00	86.86 ± 0.30 (85.60 to 87.30)
Nigeria/1975 lineage II	0.7	31.52	10:15 12:30 16:45	
Dorcas UAE/1986 lineage III	0.5	32.10	12:30 12:30 18:00	
Iran/2011 lineage IV	0.3	33.48	18:30 13:00 15:30

**Table 5:** Inter-assay repeatability of PPRV RT-LAMP assay performed on serial dilutions of PPRV/ Georgia/ Tbilisi 2016 isolate.

PPRV/ Georgia/ Tbilisi 2016	Mean C_T_ value	Time to positivity t_p_ [sec]			Mean t_p_ [mm:ss]	σ	%CV
		Analyst 1	Analyst 2	Analyst 3			
Neat	16.83	375; 390; 390	390; 390; 390	360; 375; 360	06:20	12.99	3.4
10^−1^	29.38	435; 435; 420	435; 435; 420	405; 405; 390	14:00	16.77	4.0
10^−2^	24.45	495; 495; 495	510; 510; 495	465; 465; 480	08:21	16.77	3.4
10^−3^	28.11	600; 600; 585	615; 585; 585	540; 540; 525	09:35	31.82	5.5
10^−4^	31.43	645; 630; 660	630; 630; 615	630; 555; 570	10:18	34.19	5.5
10^−5^	34.73	825; 825; 1005	990; 840; 1080	1065; 1020; 990	16:00	102.29	10.7
10^−6^	37.59	ND	ND	ND	N/A	N/A	N/A

ND - not detected, N/A - not applicable.

#### 3.2.4 Repeatability

The PPRV RT-LAMP assay demonstrated good repeatability and reproducibility, with the intra-assay %CV ranging from 0 to 6.69 (Table 3) and the inter-assay %CV ranging from 3.4 to 5.5 (Table 5) in the reliable detection range. For the last detectable dilution, the %CV increased and exceeded the recommended 10% criterion for a diagnostic assay (Table 5).

#### 3.2.5 Recent PPRV international outbreaks

The sensitivity of the RT-LAMP assay, relative to the real-time RT-PCR, was 96.7% for all samples tested (Table 6). Sample 1-2019/02 was negative using the real-time RT-PCR assay but positive using the RT-LAMP assay. Further investigation indicated the presence of inhibitors in this lung sample, which affected the performance of the real-time RT-PCR assay but not the RT-LAMP assay (Table 6). The remaining real-time RT-PCR negative samples (Table 6), were free of inhibitors as determined based on the performance of internal process control, and were also negative using the RT-LAMP assay. This indicated that the PPRV RT-LAMP assay gave a correct negative result for a number of specimens collected from ovines (lung, liver, tongue, swabs, spleen, blood, heart, and intestine) and caprines (blood, swabs, lung, and ganglion).

**Table 6:** Real-time RT-PCR and the RT-LAMP results in recent PPRV outbreaks.

Sample Name	Sample type	Real-time RT-PCR C_T_ value	Time to positivity t_p_ [mm:ss]	Anneal temperature T_a_ [°C]
1-2019/01	Ovine, lung	23.21	9:00	86.10
1-2019/02	Ovine, lung	ND	10:45	85.80
1-2019/03	Ovine, lung	ND	ND	ND
1-2019/04	Ovine, liver	26.84	11:00	86.40
1-2019/05	Ovine, lung	19.59	8:45	85.80
1-2019/06	Ovine, tongue	26.13	11:00	85.80
1-2019/07	Ovine, swab	ND	ND	ND
1-2019/08	Ovine, intestine	ND	ND	ND
1-2019/09	Ovine, lung	ND	ND	ND
1-2019/10	Ovine, lung	ND	ND	ND
1-2019/11	Ovine, liver	ND	ND	ND
1-2019/12	Ovine, heart	ND	ND	ND
2-2019/01	Caprine, swab	27.63	ND	ND
2-2019/02	Caprine, swab	ND	ND	ND
2-2019/03	Caprine, swab	19.91	7:57	86.70
2-2019/04	Ovine, swab	26.13	11:12	86.70
2-2019/05	Ovine, swab	ND	ND	ND
2-2019/06	Caprine, liver	25.87	7:27	86.30
2-2019/07	Caprine, ganglion	ND	ND	ND
2-2019/08	Caprine, lung	ND	ND	ND
1-2016/01	Ovine, spleen	27.08	10:15	86.80
1-2016/02	Ovine, lung	21.99	07:45	86.80
1-2016/03	Ovine, spleen	28.83	12:30	86.80
1-2016/04	Ovine, Lung	30.61	12:15	86.70
1-2016/05	Ovine, Blood	ND	ND	ND
1-2016/06	Ovine, spleen	27.59	09:30	86.80
1-2016/07	Ovine, lung	28.67	11:00	86.80
1-2016/08	Ovine, Blood	33.07	ND	ND
1-2016/09	Ovine, spleen	28.88	11:45	86.90
1-2016/10	Ovine, lung	ND	ND	ND

ND – not detected.

### 3.3 PPRV animal experiment

**Fig. 3:**
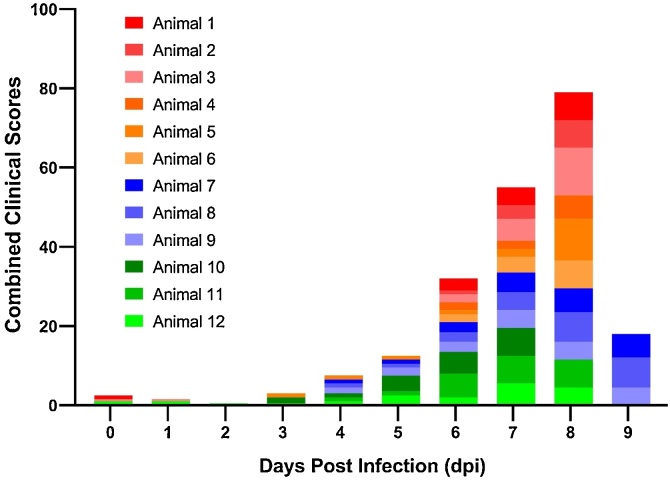
Combined clinical scores of goats (n = 12) inoculated with PPRV. Clinical score for each goat is represented by different colour.

Typical clinical signs of PPRV infection (high fever, oculo-nasal discharges, depression and apathy, diarrhea, and coughing) were observed from 7 dpi which corresponds with the increase in the combined clinical scores for all goats (Fig. 3). Although there was also some increase in the combined clinical scores between 4 and 6 dpi, mainly due to eye redness, the clinical signs were not specific to PPR infection and it is highly unlikely that the veterinary examination would identify PPR disease.

All samples (n = 36) collected from goats prior to infection with PPRV were negative by both real-time RT-PCR and RT-LAMP assays. A total of 117 samples were collected from the PPRV-infected goats at various stages of disease (4–9 dpi). The sensitivity of the LAMP assay, relative to real-time RT-PCR, was 91.74% for all sample types tested (97.44% for EDTA bloods, 81.08% for ocular swabs and 96.97% for nasal swabs).

Considering goats sampled at 6–9 dpi, the sensitivity of the RT-LAMP assay, relative to the real-time RT-PCR assay, was 101.27% - no significant difference in sensitivity was found between both assays. EDTA blood samples (n = 27), ocular swabs (n = 27) and nasal swabs (n = 25) were tested positive using both assays: the RT-LAMP and the real-time RT-PCR. One nasal swab (8 dpi, goat 4) was tested negative by both assays, possibly indicating a sampling issue. Interestingly, one nasal swab (6 dpi, goat 1) was negative using the real-time RT-PCR assay, but positive from the RT-LAMP assay after multiple analyses. The mean C_T_ value for all sample types was 35.57, 30.40, 25.25, 22.74, and 23.20 at 2, 4, 6, 8 and 9 dpi, respectively (Table 7). The C_T_ values ranged from 17.68 to 30.91 during the 6–9 dpi in all sample types. The mean t_p_ was 11 min 15 s ranging from 07:30 (mm:ss) to 18:30 (mm:ss) by the RT-LAMP assay, when considering all sample types (n = 81) taken from animals on the 6–9 dpi. The mean T_a_ 85.21 °C ± 0.41 (85.21 °C range 84.30–86.50 °C) calculated for all sample tested from the animal experiment was significantly lower than the previously-calculated mean T_a_ of 86.86 °C ± 0.30 (Table 4, ranged 85.60 °C to 87.30 °C) during the assay validation (t-test, p < 0.005). In addition, there was a significant difference (t-test, p < 0.005) between the mean T_a_ of all blood samples (85.62 °C ± 0.26) and the mean T_a_ of ocular swabs (84.92 °C ± 0.27) and between the mean T_a_ of all blood samples (85.62 °C ± 0.26) and the mean T_a_ of nasal swabs (84.92 °C ± 0.27).

**Table 7:** Real-time RT-PCR and the RT-LAMP results from the PPRV animal experiment. The mean values were calculated from 12 animals on different days post infection.

	Blood samples		Ocular swabs		Nasal swabs	
Day post infection (dpi)	RT-PCR [Ct values] mean ± SD (range)	RT-LAMP [mm:ss] mean ± SD (range)	RT-PCR [Ct values] mean ± SD (range)	RT-LAMP [mm:ss] mean ± SD (range)	RT-PCR [Ct values] mean ± SD (range)	RT-LAMP [mm:ss] mean ± SD (range)
2	35.57*± 1.87 (34.05 to 37.66)	ND	ND	ND	ND	ND
4	**28.34 ± 1.57** (26.66 to 31.19)	**13:34 ± 2:45** (10:45 to 19:00)	**32.56 ± 4.71** (21.97 to 39.60)	**15:00 ± 1:57** (12:45 to 16:15)	**30.78 ± 3.95** (26.52 to 36.20)	**16:15 ± 3:03** (11:00 to 19:30)
6	**23.36 ± 1.11** (22.22 to 25.47)	**09:56 ± 1:27** (8:00 to 12:15)	**26.63 ± 2.84** (20.54 to 30.91)	**13:19 ± 2:23** (10:00 to 18:30)	**25.83 ± 2.60** (23.09 to 30.81)	**12:20 ± 1:21** (10:00 to 14:15)
8	**24.30 ± 1.35** (23.01 to 27.66)	**10:58 ± 1:27** (9:30 to 13:15)	**22.46 ± 2.14** (19.60 to 26.40)	**12:06 ± 1:29** (10:30 to 14:15)	**21.35 ± 2.17** (17.68 to 24.70)	**10:40 ± 1:04** (9:15 to 12:45)
9**	**24.34 ± 0.68** (23.78 to 25.11)	**9:10 ± 0:17** (9:00 to 9:30)	**22.15 ± 0.41** (21.79 to 22.60)	**8:30 ± 00:54** (7:30 to 9:15)	**23.11 ± 1.93** (21.10 to 24.95)	**8:30 ± 0:26** (8:15 to 9:00)

ND – not detected.

* only three animals were tested positive using the real-time RT-PCR.

** only three animals were still alive and available for testing.

### 3.4 Detection of PPRV in the ocular swabs from animal experiment

The mean C_T_ value detected in the ocular swabs harvested at 0, 3, 5, and 11 days post incubation at 37 °C was 25.09 ± 0.36 with a mean t_p_ of 10 m in 30 s ±56 s (T_a_ 84.3 °C ÷ 85.3 °C) which indicates that no RNA degradation had occurred in these samples. The F-gene RT-PCR assay was also performed on the same samples and characteristic 447 bp bands were visible for each time point confirming that all samples were suitable for Sanger sequencing.

## 4 Discussions

We developed the PPRV RT-LAMP assay, which is considerably cheaper in comparison with the real-time RT-PCR assay, but offers equivalent sensitivity and has the potential to be used in the field. Considering the distribution of the small ruminant population in developing countries, over 80% of the global population of sheep and goats are threatened by PPR (World Organisation for Animal Health, 2019). We recommend the implementation of the RT-LAMP assay into small-scale diagnostics laboratories in developing countries, which don’t have real-time PCR assays in place. The LAMP equipment cost is significantly cheaper and less-complicated for routine use e.g. the Gene III (OptiGene Ltd., UK) costs £6,750 whereas the Quant Studio 5 real-time PCR systems costs £25,000 (ThermoFisher). Moreover, the estimated cost per reaction was £2.4 for the RT-LAMP and £4.8 for the real-time RT-PCR assay. The limited financial resources of the veterinary services in developing countries limits the surveillance and testing regimes that can be mobilized for the control of PPRV. Therefore the implementation of accurate, rapid, cost‐effective diagnostics are critical to achieve the global PPR eradication by 2030.

In this study we found that the primers targeting the N-gene provided the most efficient RT-LAMP assay and allowed for the reaction time to be completed within 20 min. Seven primer sets, targeting genes L, F and N, were initially designed and tested based on all full-length PPRV genome sequences available in public domains, but only one primer set was suitable for further validation. Since multiple primers are required for the LAMP amplification, primer hybridisation resulting in non-specific amplifications is a common problem (Wang et al., 2015; Watts et al., 2014). To eliminate the risks of false-positives, the initial duration of the LAMP reaction was extended to 60 min in order to reject any primer set with a tendency for non-specific amplification. In addition, a number of PPRV-negative specimens collected from ovines (lung, liver, tongue, swabs, spleen, blood, and heart, intestine) and caprines (blood, nasal and ocular swabs, lung, ganglion) were analyzed using the PPRV RT-LAMP assay with no false-positive results observed.

Seven PPRV isolates originating from different geographical regions were successfully detected indicating that the RT-LAMP assay was specific for PPRV. In addition, no amplification was observed when using bluetongue virus, foot-and-mouth disease virus, sheeppox virus, goatpox virus, measles virus and phocine distemper virus as a template. In comparison with the gold-standard real-time RT-PCR assay, the diagnostic sensitivity of the RT-LAMP assay was 96.7% using samples from recent PPRV outbreaks and 101.27% using samples collected one day prior to onset of clinical symptoms in experimentally-infected animals. The LOD of the RT-LAMP assay was estimated between 0.3 and 0.8 log_10_ TCID_50_ ml^−1^ for each of the four PPRV genetic lineages which equates to a C_T_ value ranging from 31.52 to 33.48.

We found that the T_a_ was similar within each matrix when the same treatment was applied e.g. manual extraction, or automated extraction. However, the mean T_a_ differed significantly between the initial assay validation performed on dilutions of cell culture material and the samples extracted manually from the animal experiment (bloods and swabs). Additionally, there was a significant difference between the mean T_a_ between blood and swab samples extracted manually from the animal experiment. It is likely that the variation in the T_a_ could be attributed to the presence of carry over contaminants from the different buffers used (e.g. EDTA buffer, LFD buffer, PBS, cell culture media) or possibly even from the different extraction methods used. This should be given consideration as confirming the positives based on a narrow range of T_a_ may not be appropriate. For instance, annealing analysis is generally recommended since non-specific amplicons can be generated-particularly at later stages of the LAMP assay. However in this study, we have omitted primer sets which yielded false positives, reduced the assay time to less than 20 min and tested representative samples to give confidence that false positives will not occur. Therefore, there may be little benefit in performing annealing analysis once the RT-LAMP has been thoroughly-validated for a specific extraction method and matrix.

In samples from experimentally-infected animals, we demonstrated that the RT-LAMP assay had comparable sensitivity to the real-time RT-PCR assay between 6 and 9 dpi and is capable of detecting PPRV prior to clinical signs. It is important to highlight that characteristic symptoms of PPR in the experimentally-infected goats had first been observed at 7 dpi. Early in infection (4 dpi; prior to clinical signs), PPRV was detected using the RT-LAMP assay in EDTA blood and nasal and oral swabs. The performance of the RT-LAMP assay suggests that the assay could be used in isolation as an accurate and sensitive diagnostic assay for PPRV. Furthermore, all samples were manually extracted using the MagMax Core extraction kit with the magnetic separation system which is suitable for the use in the field as it does not require electrical equipment. Additional advantages of this extraction method include speed (1 sample can be extracted in ˜10 min) and the reagents can be stored at temperatures between 15 °C and 30 °C. In addition, we re-tested twelve ocular swabs collected from experimentally-infected goats at 6 dpi using the direct RT-LAMP assay and found only a small loss in sensitivity (10 out of 12 samples were positive, 83.3%). Recent advances in LAMP chemistry demonstrates robust performance even in high concentrations of amplification inhibitors (da Silva et al., 2019). Therefore, it is quite likely that the development of more robust polymerases (e.g. Bst 3.0) will facilitate direct detection of virus in field samples without any losses in sensitivity and lead to further cost savings and procedure simplification.

In this study, we also aimed to simulate the likely scenarios for which we consider the RT-LAMP assay to be best suited. Impediments to sample analysis in developing countries involve poor infrastructure, transport delays and the lack of cold chain. It was therefore important to determine the effect of adverse sample storage conditions on the detection capacity of the RT-LAMP. By using the storage buffer which is included in the PESTE-TEST lateral flow device kit, we found that PPRV RNA can be detected in ocular swabs following incubation at 37 °C for 11 days. Both secondary assays (RT-LAMP and real-time RT-PCR) yielded similar results indicating that either assay could be used as the confirmatory diagnostic. Furthermore, the samples were also suitable for sequencing which shows that the LFD kit buffer was suitable to preserve PPRV RNA for downstream analyses despite adverse storage conditions (incubation at 37 °C for 11 days). The PPRV RT-LAMP developed in this study is more sensitive in comparison to the field PESTE-TEST, which achieved an estimated sensitivity of 10^3^ TCID_50_ (Baron et al., 2014), and could be used alongside the LFD field test to either confirm positives or negate negatives in previously unaffected regions.

## 5 Conclusions

We have developed a rapid, specific, sensitive and low-cost RT-LAMP assay for the detection of PPRV. The sensitivity and specificity of this assay is comparable with the “gold standard” real-time RT-PCR assay. The PPRV RT-LAMP assay has the capacity to enable rapid field diagnosis which if utilized in affected countries, will contribute to the global eradication effort of PPR.
