# Supplementary material for: A rapid RT-LAMP assay for the detection of all four lineages of Peste des Petits Ruminants Virus
Source: J Virol Methods. 2019 Dec;274:113730. doi: 10.1016/j.jviromet.2019.113730 (PMC6859475; doi:10.1016/j.jviromet.2019.113730)
Supplement: Supplementary file 1 [file mmc1.docx]

Table S1 (supplementary material) LAMP primer sets - design and evaluated in this study.

| **Oligo sequence (5' to 3')** | **Position** | **Oligo name** | **Primer set** |
| --- | --- | --- | --- |
| TATATGCCACAAGAGGAGGT | 552 | L_F3_a | A |
| CCAATCCTTCAGTGATGGAAT | 1003 | L_B3_a |  |
| CATGGCTGTCTCCGTCATAAGTCGAGACCTCGTTGTGATAATTGA | 735; 620 | L_FIP_a |  |
| TGGGATCTTATTGATGGGTTCTTCCCCTGAAGTTGAAGATAAGCCA | 784; 883 | L_BIP_a |  |
| CATCACAATACATAAGGACCAGC | 700 | L_LF_a |  |
| TGTTGCTCTGCTTGAACCA | 837 | L_LB_a |  |
| ACTCTAAGATAAGTCACCAGGT | 350 | L_F3_b | B |
| ATGGCTGTCTCCGTCAT | 734 | L_B3_b |  |
| CCTCTTGTGGCATATATGGGACTGGCACATGCATAGCTCTCA | 567; 462 | L_FIP_b |  |
| GGGATGCATTCGAGGTGCTTCACAATACATAAGGACCAGC | 593; 698 | L_BIP_b |  |
| CTCTGTCTTGATAGTGAACCAGAA | 522 | L_LF_b |  |
| CGAGACCTCGTTGTGATAATTG | 619 | L_LB_b |  |
| GGTGGAGTTTGTTACCTGG | 4263 | L_F3_c | C |
| GCCATGAATCGGCTCAAT | 4659 | L_B3_c |  |
| TGTTGATGTCGTCATCGCCAAACAGCAATGTCCATGATTGA | 4408;4318 | L_FIP_c |  |
| CAGTGTGCTGCCATCAATTGGTCCTTTGCTCATCCGAGA | 4465;4572 | L_BIP_c |  |
| GCGGCTATTTCATTCATGTGAT | 4382 | L_LF_c |  |
| GGGCAAGTACCAGATGGG | 4515 | L_LB_c |  |
| GCAAATTGTGCGTCAGTYY | 1150 | F_F3_d | D |
| YGAGCCCTGGGTTGATT | 1594 | F_B3_d |  |
| CCTAAATTGGTGCCTACATCCAGTARGTGGATGGAGTGACRA | 1379;1259 | F_FIP_d |  |
| ACAAGACTGGAGAATGCAAAGGAGCTGCCAGTGCTATGTAC | 1390;1493 | F_BIP_d |  |
| TCGACTGCCGACCTGTA | 1293 | F_LF_d |  |
| GCTCCTAGATGCATCAGACC | 1413 | F_LB_d |  |
| CCAACCTTATCTGAGATCAAGG | 844 | F_F3_e | E |
| GTATTGTCACTCCATCCACC | 1279 | F_B3_e |  |
| TACAACGCATTCTGGCTGCACCCTAAATATGTAGCCACTCAG | 1034; 933 | F_FIP_e |  |
| CCATTGCTTCAGGAATGTTTCAGGAAACTGACGCACAATTTGC | 1045; 1168 | F_BIP_e |  |
| CTCTGGAGTGAATACGCATGA | 1005 | F_LF_e |  |
| AATCGTGCGCCAGAACC | 1079 | F_LB_e |  |
| TGTTAGCCTCCATACTAGCA | 497 | N_F3_f | F |
| TGTCAATGTCGCAGATCATT | 775 | N_B3_f |  |
| TGTCAAGGCGAAATTCCCCAAAGAACTGAGAAGGTGGGTTA | 637; 573 | N_FIP_f |  |
| CGGCGGTTCATGGTATCTCTCCAATCCTTGGCTTGTTGC | 688; 751 | N_BIP_f |  |
| TCACTCTCCTTTGTTGTGTGT | 616 | N_LF_f |  |
| ATACTTGACATCAAGAGGACCC | 709 | N_LB_f |  |
| GTTATCATAGTCCCGATTCCC | 100 | N_F3_g | G |
| ACGTGATGCAAAGGTCAA | 357 | N_B3_g |  |
| CCGTTGATGTCAGGATCTCCGCCGTTCAAGACTGCTCG | 197; 141 | N_FIP_g |  |
| AGTCACCCGGGCAATTGATACCGGACTGGGACCTAGTAC | 245; 337 | N_BIP_g |  |
| GCCAATCTGACAAGCCTGT | 176 | N_LF_g |  |
| CCGCCTTGTTGAGGTAGTT | 297 | N_LB_g |  |
