# Supplementary material for: A rapid RT-LAMP assay for the detection of all four lineages of Peste des Petits Ruminants Virus
Source: J Virol Methods. 2019 Dec;274:113730. doi: 10.1016/j.jviromet.2019.113730 (PMC6859475; doi:10.1016/j.jviromet.2019.113730)
Supplement: Supplementary file 2 [file mmc2.docx]

Table S2 (supplementary material). Specificity of RT-LAMP assay.

| **Sample Name** | **Pathogen** | **Time to positivity t_p_ (mm:ss)** | **Anneal temperature T_a_ [°C]** |
| --- | --- | --- | --- |
| PPRV/Guinea/1988 lineage I | PPRV | 8:30 | 86.4 |
| PPRV/Guinea/1988 lineage I | PPRV | 7:45 | 86.5 |
| PPRV/Ivory Coast/1997 lineage I | PPRV | 7:00 | 86.6 |
| PPRV/Ivory Coast/1997 Lineage I | PPRV | 7:30 | 86.5 |
| PPRV/Ghana/1978/1 Lineage II | PPRV | 7:30 | 86.6 |
| PPRV/Ghana/1978/1 Lineage II | PPRV | 7:15 | 86.6 |
| PPRV/Nigeria/1975/2 Lineage II | PPRV | 7:30 | 86.1 |
| PPRV/Nigeria/1975/2 Lineage II | PPRV | 7:30 | 86.2 |
| PPRV/Dorcas U.A.E./1986 Lineage III | PPRV | 8:15 | 85.7 |
| PPRV/Dorcas U.A.E./1986 Lineage III | PPRV | 8:30 | 85.7 |
| PPRV/Iran/2011 Lineage IV | PPRV | 5:45 | 86.3 |
| PPRV/Iran/2011 Lineage IV | PPRV | 6:00 | 86.3 |
| PPRV/Georgia/Tbilisi/2016 Lineage IV | PPRV | 5:45 | 86.7 |
| PPRV/Georgia/Tbilisi/2016 Lineage IV | PPRV | 5:30 | 86.7 |
| Morbillivirus PDV | PDV | ND | - |
| Morbillivirus MeV | MeV | ND | - |
| BTV016/19 Well 1A (Pos Con 170717) BTV-8 | BTV | ND | - |
| BTV Serotype BTV-1 | BTV | ND | - |
| BTV Serotype BTV-2 | BTV | ND | - |
| BTV Serotype BTV-3 | BTV | ND | - |
| BTV Serotype BTV-4 | BTV | ND | - |
| BTV Serotype BTV-5 | BTV | ND | - |
| BTV Serotype BTV-6 | BTV | ND | - |
| BTV Serotype BTV-7 | BTV | ND | - |
| BTV Serotype BTV-8 | BTV | ND | - |
| BTV Serotype BTV-9 | BTV | ND | - |
| BTV Serotype BTV-10 | BTV | ND | - |
| BTV Serotype BTV-11 | BTV | ND | - |
| BTV Serotype BTV-12 | BTV | ND | - |
| BTV Serotype BTV-13 | BTV | ND | - |
| BTV Serotype BTV-14 | BTV | ND | - |
| BTV Serotype BTV-15 | BTV | ND | - |
| BTV Serotype BTV-16 | BTV | ND | - |
| BTV Serotype BTV-17 | BTV | ND | - |
| BTV Serotype BTV-18 | BTV | ND | - |
| BTV Serotype BTV-19 | BTV | ND | - |
| BTV Serotype BTV-20 | BTV | ND | - |
| BTV Serotype BTV-21 | BTV | ND | - |
| BTV Serotype BTV-22 | BTV | ND | - |
| BTV Serotype BTV-23 | BTV | ND | - |
| BTV Serotype BTV-24 | BTV | ND | - |
| BTV Serotype BTV-25 | BTV | ND | - |
| BTV Serotype BTV-26 | BTV | ND | - |
| BTV Serotype BTV-27 | BTV | ND | - |
| BTV Serotype BTV-29 | BTV | ND | - |
| MAY 1/2017 FMDV Sample 1 | FMDV | ND | - |
| MAY 1/2017 FMDV Sample 2 | FMDV | ND | - |
| MAY 1/2017 FMDV Sample 3 | FMDV | ND | - |
| MAY 1/2017 FMDV Sample 4 | FMDV | ND | - |
| MAY 1/2017 FMDV Sample 5 | FMDV | ND | - |
| Sheeppox virus | SPPV | ND | - |
| Goatpox virus | GTPV | ND | - |
